# Characteristics and outcomes of adult patients with asthma presenting with COVID-19: A comparative cohort study

**DOI:** 10.5339/qmj.2023.15

**Published:** 2023-08-08

**Authors:** Eman Alhmoud, Raja Barazi, Mohammed Saad, Dania Al Khiyami, Reem El Ajez, Dana Bakdach, Ali Omrani, Wanis Ibrahim, Rasha El Anany, Moza Al-Hail

**Affiliations:** ^1^Pharmacy Department, Hamad Medical Corporation, Doha, Qatar. E-mail: ealhamoud@hamad.qa ORCID: 0000-0002-3871-7088; ^2^Department of Medicine, Hamad Medical Corporation, Doha, Qatar; ^3^College of Medicine, Qatar University, Doha, Qatar; ^4^Weill Cornell Medicine - Qatar (WCM-Q), Qatar

**Keywords:** Asthma, COVID-19, morbidity, mortality

## Abstract

Background: Bronchial asthma affects about 20% of Qatar’s population. The impact of asthma on COVID-19 outcomes is controversial. The aim of this study was to explore the impact of asthma on COVID-19 outcomes and the predictors of COVID-19-related morbidity and mortality in a cohort of asthma patients infected by COVID-19.

Methods: This is a retrospective cohort study of adult patients with asthma infected with COVID-19, who were recruited from Hamad Medical Corporation (HMC), the main healthcare system in Qatar. Patients were matched to a control group of non-asthmatic COVID-19 patients (1:2) based on sex, age, and other comorbidities.

Results: Between March and August 2020, 616 patients with asthma met the inclusion criteria. The need for hospitalization among patients with asthma was independently associated with older age (adjusted odds ratio [aOR] for 10 years, 1.32; 95% confidence interval [CI], 1.13–1.54; *p* = 0.001) and hypertension (aOR, 2.4; 95% CI, 1.43–3.93; *p* = 0.001) but not with the use of inhaled corticosteroids (ICS), long-acting beta2 agonists, montelukast, or tiotropium. Patients with asthma required less hospitalization for COVID-19 than non-asthmatic patients (28.2% vs. 37.3%, respectively; aOR, 0.59; 95% CI, 0.77–0.90; *p* < 0.001). However, admission to the intensive care unit (ICU) was comparable between both groups (3.3% vs. 2.2%; aOR, 1.64; 95% CI, 0.78–3.43; *p* = 0.193).

No difference in mortality rate was observed between the two groups.

Conclusions: In Qatar, adult patients with asthma do not appear to be at higher risk of COVID-19-related hospitalization or ICU admission compared to the general adult COVID-19-infected population. Older age and hypertension were the only significant predictors of COVID-19-related hospitalization among patients with asthma. Further larger studies are required to confirm such an association.

## Background

In 2020, the U.S. Centers for Disease Control and Prevention identified asthma patients at high risk for hospitalization and other severe outcomes from COVID-19.^1^ The severity of COVID-19 in patients with asthma depends on several factors, including the underlying severity of asthma, the phenotype of asthma, other comorbidities, and asthma medications.^2^ The use of inhaled corticosteroids (ICS) undoubtedly reduces the rate of asthma exacerbations, but their effect on COVID-19-infected patients is yet to be explored. The airway angiotensin-converting enzyme 2 (ACE2) receptor expression, which SARS-CoV-2 utilizes to enter host cells, is found to be increased in patients with asthma maintained on long-term ICSs.^3,4^ Consequently, the need for ICS use during COVID-19 infection and their impact on outcomes are areas of debate. Furthermore, patients with asthma have impaired production of antiviral interferon, and this makes their innate immune system vulnerable to lower-respiratory-tract infections. Therefore, patients with asthma are believed to be at high risk of COVID-19 morbidity and mortality.^5^

On the contrary, the prevalence of COVID-19 infection among patients with chronic lung disease (including asthma) was observed to be lower than that in the general population.^6^ A study by Mahdavin et al. revealed that there was no causal relation between the history of asthma and the rate of acute respiratory distress syndrome or hospitalization.^7^ In fact, Hernandez-Galdamez et al. and Santos et al. suggested that asthma was a protective factor against death.^8,9^ Moreover, studies conducted by Yehia et al. and Siso-Almirall et al. indicated that asthma was not a predictor of COVID-19 mortality.^10,11^ Current evidence delineates that asthma is not in the top 10 comorbidities associated with COVID-19-related poor outcomes or fatalities.^12–14^ Conversely, asthma was associated with an increased risk of death in patients with COVID-19 in a study by Almazeedi et al.^15^ Thus, the impact of COVID-19 on people with asthma remains uncertain. Considering that 19.8% of the Qatari population has asthma,^16^ it is important to understand the impact of premorbid asthma diagnosis on COVID-19 infection severity and outcome.

The primary aim of this study was to investigate COVID-19 outcomes and their predictors in a cohort of unvaccinated COVID-19-infected patients with asthma. The secondary aim was to compare COVID-19-related outcomes between patients with and without a diagnosis of asthma. Outcomes evaluated included the need for hospitalization, the need for admission to an intensive care unit (ICU), and in-hospital mortality.

## Methods

### Study design and participants

This was a retrospective cohort study. Adult patients (aged ≥18 years) with a history of asthma and laboratory-confirmed COVID-19 infection (positive result using real-time polymerase chain reaction testing of a nasal or pharyngeal swab) for more than 5 months (March 10 to August 10, 2020) were included. Patients with asthma were identified through electronic medical records (Cerner®, Cerner Corporation, Kansas City, MO) using the diagnosis codes coupled with the pharmacy records of asthma medication refills. Diagnosis of asthma was determined by reviewing the physicians’ notes for each patient. Patients with no refills of asthma-related therapies in the last year before the study were excluded. The outcome of COVID-19-related hospitalization was counted as admission to acute care facilities but not to quarantine facilities. The decisions to admit patients to acute care facilities were made based on the Hamad Medical Corporation (HMC) institutional protocol for the management of COVID-19. ICU admission and mortality information was obtained from electronic medical records. To investigate the impact of underlying asthma on COVID-19 outcomes, patients with asthma were matched to patients without asthma from the national database of COVID-19 cases, based on sex, age (within a 5-year margin), diabetes, hypertension, chronic kidney disease, chronic liver disease, and coronary artery disease, as these were previously found to be associated with worse COVID-19 outcomes.^13,14^ The ratio of asthmatic to non-asthmatic patients was 1:2.

This study was conducted across HMC, the main emergency and tertiary healthcare provider in Qatar, where all COVID-19-diagnosed patients in the country, including those seen in governmental and private health centers, were registered. The study was approved and conducted in compliance with the requirements of the Institutional Review Board/Human Subjects Research Committee of the Medical Research Center, Hamad Medical Corporation, Qatar (MRC-01-20-795).

## Statistical Analysis

No statistical sample size calculation was performed a priori. All patients meeting the eligibility criteria were included. Continuous variables were presented as median (interquartile range [IQR]), and categorical variables were presented as counts (percentages). Characteristics and outcomes of patients with asthma who required hospitalization were compared with those who did not require hospitalization. To explore the risk factors independently associated with hospitalization for COVID-19 among patients with asthma, a multivariable logistic regression model was used. Multiple imputations were used to complete missing data on weight (16 observations). The ratio of asthmatic to non-asthmatic patients was 1:2; this matching was performed using the “calipmatch” Stata package. Conditional logistic regression was used to estimate the adjusted odds ratio [aOR] of COVID-19-related hospitalization and ICU admission in patients with asthma compared to matched non-asthmatic patients. Due to the residual difference in age after matching, age was included in the conditional logistic regression model. To assess the robustness of the results, the effect of asthma on COVID-19-related hospitalization and ICU admission was reinvestigated using an unconditional logistic regression model including all patients (with and without asthma), adjusting for the previously mentioned matching variables. Two-sided *p*-values <0.05 were considered statistically significant, and 95% confidence intervals (CI) were reported for estimates of aOR from logistic regression models. Statistical analyses were performed using Stata (StataCorp. 2021. Stata Statistical Software: Release 17. College Station, TX: StataCorp LLC.).^16^

## Results

### Findings of asthma cohort

Over the study period, 616 patients with asthma met the inclusion criteria. About half of them were women (52%), and the median age was 44 years. Forty-four percent of patients were on ICSs, 42% were on LABA, 18% were on montelukast, and 2.9% were on tiotropium. One patient was receiving oral corticosteroids (OCS) for a long period, and two patients were maintained on biological agents. Thirty-nine percent required hospitalization for COVID-19, with a median hospital stay of 10 days (IQR, 5–15). Table 1 presents the baseline characteristic comparison between asthmatic and non-asthmatic patients.

Multiple logistic regression demonstrated that the need for hospitalization among patients with asthma was independently associated with older age (aOR, 1.32, for every 10-year increase in age; 95% CI, 1.13–1.54; *p* = 0.001) and hypertension (aOR, 2.4; 95% CI, 1.43–3.93; *p* = 0.001) but not with the use of ICSs (regardless of dose), LABA, montelukast, or tiotropium (Table 2).

Overall, hospitalized patients were significantly older, had higher weight, had additional comorbidities, and were originally from different World Health Organization regions compared to non-hospitalized patients (Table 3). About 8% of patients with asthma required ICU admission due to COVID-19, of whom 4.2% required invasive mechanical ventilation. Moreover, 2.6% of patients with asthma died during follow-up (up to 60 days from the date of diagnosis of COVID-19 infection).

### Findings of the matched cohorts (asthma vs. no-asthma diagnosis)

Considering individual matching on multiple variables, 454 asthmatic patients were matched to 908 non-asthmatic patients based on sex, age, and other previously described comorbidities. In the matched cohort, significantly fewer asthmatic patients (28%) required hospitalization for COVID-19 compared to non-asthmatic patients (37%) (aOR, 0.59; 95% CI, 0.77–0.90; *p* < 0.001). However, 3.3% of patients with asthma required ICU admission compared to 2.2% of patients without asthma (aOR, 1.64; 95% CI, 0.78–3.43; *p* = 0.193), a difference that was not statistically significant. Two asthmatic patients died because of COVID-19 compared to one non-asthmatic patient (*p* = 0.26). When sensitivity analysis was conducted using unconditional logistic regression on all patients within the COVID-19 national registry (*N* = 5616) with adjustment for sex, age, diabetes, hypertension, chronic kidney disease, chronic liver disease, coronary artery disease, and cancer, asthma was still confirmed to be independently associated with less hospitalization to COVID-19 (aOR, 0.61; 95% CI, 0.49–0.76; *p* < 0.001) but not with COVID-19-related ICU admission (aOR, 1.48; 95% CI, 0.94–2.34; *p* = 0.09).

## Discussion

In this retrospective cohort study, we described the clinical characteristics and outcomes of asthma patients infected with COVID-19, explored the characteristics associated with hospitalization for COVID-19, and compared COVID-19 outcomes between asthmatic and non-asthmatic patients. The association between asthma and COVID-19 outcomes has been inconsistently described in the literature since the beginning of the pandemic.^12–15^ During the first year of the pandemic, many studies have suggested an increased risk of severe COVID-19 infections among asthmatic patients. However, most of those studies were mainly conducted among hospitalized patients, rather than also including community patients, thus potentially biasing the risks.^15,17–19^ On the contrary, many community-based cohorts found no association between asthma and poor COVID-19-related outcomes, including hospitalization, ICU admission, or death.^20^ Further understanding of the disease demonstrated that worse outcomes were mostly attributed to other concomitant comorbidities among this population. In 2021, Izquierdo et al. analyzed the clinical data of more than 70,000 COVID-19-infected patients with asthma before the introduction of COVID-19 vaccines. Comorbidities, including hypertension, obesity, diabetes, and dyslipidemia, were associated with severe COVID-19 disease.^21^ This is similar to that found in our cohort as hypertension and older age were independently associated with COVID-19-related hospitalization.

In our study, a history of asthma exacerbation was not associated with hospitalization for COVID-19. However, a previous South Korean study found an association between death and a history of acute exacerbation of asthma within 1 year of COVID-19 infection.^22^ Similarly, Dolby et al. showed a similar association when OCS was utilized as an exposure variable for exacerbation risk.^23^ Moreover, a recent cohort study from Scotland demonstrated that a history of asthma attacks, defined by a prescription of two or more courses of OCSs or asthma hospitalization in the preceding 2 years, increased the risk of both COVID-19 hospitalization and ICU admission or death compared with those without asthma.^24^ Among patients who utilized two or more courses of OCSs, the adjusted hazard ratios (aHR) and 95% CI for COVID-19 hospitalization and ICU admissions or deaths were 1.37, 1.26–1.48 and 1.27, 1.09–1.48, respectively. Similarly, aHR and 95% CI for COVID-19 hospitalization and ICU admissions or deaths for patients with prior asthma hospitalization were 3.01, 2.59–3.49 and 2.24, 1.56–3.20, respectively.

Although we have not directly assessed the history of asthma control among our cohort, fewer patients were on OCSs or high-dose ICSs, which might explain the reported difference. Yet our smaller sample size compared to the other cohorts might have also limited the observation.

In a recent prospective cohort study utilizing data from the UK Biobank before the introduction of COVID-19 vaccination, hospitalization risk was found to be associated with asthma phenotype. The study demonstrated that nonallergic asthma and asthma with concomitant chronic obstructive pulmonary disease (COPD) were significantly associated with increased risk of hospital admission for severe COVID-19 when compared to patients without asthma or COPD, after adjustment for potential confounders, including patient’s age, sex, body mass index, and comorbidities.^25^ Only 2.4% of patients with asthma in our study had concomitant COPD, which was not found to predict the severity of COVID-19. Furthermore, data on asthma phenotypes were not captured in the current study.

The use of ICSs or LABA for asthma was not associated with hospitalization for COVID-19 in our cohort, a finding that is consistent with previous studies that did not confirm an association between the use of LABA and/or ICS and COVID-19 hospital admission, ICU admission, and mortality in patients with asthma.^19,26,27^ On the contrary, Schultze et al. found that the risk of COVID-19-related mortality was associated with the use of high-dose ICSs but not low- or medium-dose ICSs among asthma patients,^28^ raising the possibility of an association between uncontrolled asthma (indicated by increased steroid requirements) and worse outcomes. Recently, it was shown that the expression of COVID-19 infection mediators, namely ACE2 and transmembrane protease serine 2, decreased in asthma patients on ICS,^29^ a finding that supported the hypothesis of ICS protective effect against COVID-19 infection and morbidity. However, a recent systematic review^30^ concluded that among unvaccinated patients, there seemed to be moderate certainty evidence that ICSs reduced the risk of hospitalization. Nevertheless, it is worth noting that the conclusion was mainly driven by two smaller trials, whereas the largest trial that included outpatients at risk of severe disease did not show such a benefit. Moreover, no mortality benefit was identified by any other included trials. Given the mixed benefits of ICSs with such regard, the Infectious Diseases Society of America still recommends against ICSs for the management of COVID-19 unless used in the context of a clinical trial.^31^ Considering the higher reported compliance with controller medications during the pandemic, the speculated benefits of ICSs among people with asthma could have been potentially amplified because it could have contributed to a decrease in asthma exacerbations and hospitalizations. In the United States, one study revealed a 14% relative increased adherence to daily controller medications in asthma and COPD patients during the COVID-19 pandemic.^32^ In the current study, less than half of patients with asthma were receiving ICSs (44%). Although data on adherence were not reported, ICS use was not associated with the risk of hospitalization among those patients.

Our finding of lower hospitalization risk among patients with asthma compared to the control group is consistent with previous studies. In a pooled analysis of 11 epidemiological studies, asthma was reported in only 1.20% of COVID-19 hospitalized patients. Compared with the general population through comorbidity matching, a diagnosis of asthma resulted in a significantly lower risk of COVID-19 hospitalization (relative risk (RR): 0.86).^33^ Authors attributed the finding to the protective effects of ICSs against COVID-19. Similarly, Sunjaya et al. conducted a systematic review to characterize the risks of severe illness from COVID-19 among patients with asthma and found that hospitalization for COVID-19 was reduced by 13% among asthmatic patients compared to non-asthmatic cohorts.^34^ However, an update of the aforementioned review demonstrated a trend of increased hospitalization and ICU admission among asthmatic compared to non-asthmatic patients; yet the differences were not statistically significant.^35^ The reviews did not confirm whether COVID-19-vaccinated subjects were enrolled and had significant statistical heterogeneity. Different possible factors could explain the lower hospitalization and trend of increased need for intensive care support observed among patients with asthma in our study. Decreased hospitalizations might be explained by the fact that patients with asthma have a higher threshold for seeking immediate care once they develop symptoms due to the chronic nature of their respiratory symptoms. Avoidance of viral exposure in healthcare facilities is another proposed explanation, especially during the early period of the pandemic, when people feared visits to healthcare facilities.^36,37^ On the contrary, once admitted, healthcare providers may exhibit a lower threshold for admitting patients with asthma to critical care units in anticipation of their higher risk of quick deterioration. This is supported by the findings of Bloom et al., where the decision to provide critical care was not associated with clinical severity upon admission.^38^

This study has some limitations. First, the included study period was limited to the first 6 months of the pandemic. This was mostly driven by the limitation of the study approval duration coupled with the available registry for matching. Extending the study period might have identified more patients with asthma and thus have resulted in a larger studied population. However, the advantage of such a cohort still exists in the fact that all included patients were still not vaccinated and thus helped in reducing the confounding effects of the vaccines on different COVID-19 outcomes. Second, given the retrospective nature of the study, along with the use of electronic health records to obtain the studied sample, misclassification and incomplete documentation issues are unavoidable and could have some impact on the internal validity of the study. Moreover, different studies have recently coupled the phenotype of asthma to COVID-19 severity. However, in our study, we have not recorded the different phenotypes of asthma, nor the degree of asthma control, which limited our ability to investigate such correlations. Finally, the lack of identification of ethnic origin was another limitation of our study, considering recent evidence suggesting lower hospitalization risk among patients of white ethnicity compared to other ethnic groups.^39,40^ Future collaborative multicenter research is still required to have a better understanding of this observation.

## Conclusion

Based on the results of the current study, older age and history of hypertension appear to be independently associated with COVID-19 hospitalization among patients with asthma. Patients with asthma had a lower risk of hospitalization, but not ICU admission, compared to non-asthmatic patients. Further research is warranted to explore the effects of different asthma phenotypes, ethnic backgrounds, and vaccination status on the outcomes of COVID-19 infections among this patient group.

### Conflict of Interest

The authors declare that there is no conflict of interest or financial disclosures to be disclosed.

## Figures and Tables

**Table 1.** Baseline characteristic comparison between asthmatic and non-asthmatic patients.

	**Non-asthma**	**Asthma**	***p*-Value**
	***n* = 908**	***n* = 454**	
Age (year); median (IQR)	36.5 (29–47)	39 (31–49)	0.006
Female	366 (40.3%)	183 (40.3%)	1.00
WHO region			
Africa	32 (3.5%)	6 (1.3%)	<0.001
Americas	7 (0.8%)	0 (0.0%)	
Eastern Mediterranean	314 (34.6%)	261 (57.5%)	
Europe	13 (1.4%)	1 (0.2%)	
Southeast Asia	483 (53.2%)	160 (35.2%)	
Western Pacific	59 (6.5%)	26 (5.7%)	
Diabetes mellitus	134 (14.8%)	67 (14.8%)	1.00
Hypertension	130 (14.3%)	65 (14.3%)	1.00
Coronary artery disease	18 (2.0%)	9 (2.0%)	1.00
Chronic kidney disease	10 (1.1%)	5 (1.1%)	1.00
End-stage renal disease	3 (0.3%)	2 (0.4%)	1.00
Chronic liver disease	0 (0.0%)	0 (0.0%)	1.00
Cancer	0 (0.0%)	0 (0.0%)	1.00

Data are presented as n (%) unless noted otherwise. IQR: interquartile range, WHO: World Health Organization.

**Table 2.** Association of baseline characteristics with hospitalization for COVID-19 among patients with asthma.

**Characteristic**	**Adjusted odds ratio**	**95% confidence interval**	***p*-Value**
Age (10-year increase)	1.32	1.13–1.54	0.001
Weight (10-kg increase)	1.10	0.99–1.22	0.074
Male	0.73	0.5–1.07	0.104
Diabetes	1.24	0.76–2	0.388
Hypertension	2.37	1.43–3.93	0.001
Chronic kidney disease	2.79	0.95–8.18	0.061
Coronary artery disease	2.13	0.92–4.93	0.079
ICS	1.15	0.36–3.68	0.818
Tiotropium	1.12	0.37–3.37	0.844
LABA	0.82	0.25–2.68	0.745
Montelukast	0.87	0.52–1.47	0.611
Recent OCS for asthma within 3 months	1.11	0.52–2.34	0.789
Asthma ED visits in the past year	1.01	0.65–1.59	0.948

ED: emergency department, ICS: inhaled corticosteroids, LABA: long-acting beta2 agonists, OCS: oral corticosteroids.

**Table 3.** Comparison between hospitalized and non-hospitalized patients with asthma.

	**Hospitalization for COVID-19**		***p*-Value**
	**No (*n* = 379)**	**Yes (*n* = 237)**	
Age (years), median (IQR)	40 (31–49)	55 (40–64)	<0.001
Female	179 (47.2%)	141 (59.9%)	0.002
Weight (kg), median (IQR)	77 (66.8–87.4)	80 (69.4–92)	0.028
WHO region			
Africa	6 (1.6%)	1 (0.4%)	0.003
Americas	0 (0.0%)	4 (1.7%)	
Eastern Mediterranean	230 (60.7%)	160 (67.5%)	
Europe	2 (0.5%)	2 (0.8%)	
Southeast Asia	124 (32.7%)	52 (21.9%)	
Western Pacific	17 (4.5%)	18 (7.6%)	
Comorbidities			
Diabetes	63 (16.6%)	104 (43.9%)	<0.001
Hypertension	62 (16.4%)	129 (54.4%)	<0.001
Coronary artery disease	9 (2.4%)	37 (15.6%)	<0.001
Heart failure (with reduced or preserved ejection fraction)	6 (1.6%)	19 (8.0%)	<0.001
Atrial fibrillation	3 (0.8%)	17 (7.2%)	<0.001
Stroke or transient ischemic attack	2 (0.5%)	9 (3.8%)	0.003
Peripheral arterial disease	1 (0.3%)	4 (1.7%)	0.055
Dementia	2 (0.5%)	4 (1.7%)	0.15
COPD	1 (0.3%)	14 (5.9%)	<0.001
Interstitial lung disease or lung fibrosis	1 (0.3%)	3 (1.3%)	0.13
Cystic fibrosis	0 (0.0%)	1 (0.4%)	0.21
Bronchiectasis	0 (0.0%)	1 (0.4%)	0.2
Connective tissue disease	6 (1.6%)	2 (0.8%)	0.43
Chronic kidney disease	5 (1.3%)	26 (11.0%)	<0.001
On dialysis before COVID-19	0 (0.0%)	5 (2.1%)	0.005
Chronic liver disease	0 (0.0%)	2 (0.8%)	0.073
Solid organ transplantation	1 (0.3%)	5 (2.1%)	0.023
ICS daily dose category			
High	22 (5.8%)	25 (10.5%)	0.18
Medium	89 (23.5%)	50 (21.1%)	
Low	49 (12.9%)	37 (15.7%)	
No ICS	219 (57.8%)	125 (52.7%)	
Recent OCS for asthma within 3 months	25 (6.6%)	17 (7.2%)	0.78
ED visits for asthma in the past year			
None	280 (73.9%)	181 (76.4%)	0.21
1	78 (20.5%)	37 (15.6%)	
≥2	21 (5.6%)	19 (8%)	
Hospitalization for asthma in the past year			
None	359 (94.7%)	215 (90.7%)	0.19
1	16 (4.2%)	17 (7.2%)	
≥2	4 (1.1%)	5 (2.1%)	

COPD: chronic obstructive pulmonary disease, ED: emergency department, ICS: inhaled corticosteroid, IQR: interquartile range, OCS: oral corticosteroid, WHO: World Health Organization.
